# Considerations for the use of plasma cytokeratin 18 as a biomarker in pancreatic cancer

**DOI:** 10.1038/sj.bjc.6605494

**Published:** 2010-01-05

**Authors:** C Dive, R A Smith, E Garner, T Ward, S St George-Smith, F Campbell, W Greenhalf, P Ghaneh, J P Neoptolemos

**Affiliations:** 1 Clinical and Experimental Pharmacology Group, Paterson Institute for Cancer Research, University of Manchester, Manchester, UK; 2 Liverpool Experimental Cancer Medicines Centre and NIHR Pancreas Biomedical Research Unit, Royal Liverpool University Hospital, 5th Floor UCD Building, Daulby St, Liverpool L69 3GA, UK; 3 Department of Pathology, Royal Liverpool University Hospital, Prescot St, Liverpool L7 8XP, UK

**Keywords:** pancreatic cancer, cytokeratin 18, circulating biomarker, M65, M30, necrosis, apoptosis

## Abstract

**Background:**

Enzyme-linked immunoassays of full-length (M65) and/or caspase-cleaved (M30) cytokeratin 18 (CK18) released from epithelial cells undergoing necrosis and/or apoptosis, respectively, may have prognostic or predictive biomarker utility in a range of solid tumour types. Characterisation of baseline levels of circulating full length and cleaved CK18 specifically in patients with pancreatic cancer.

**Methods:**

Plasma samples from 103 patients with pancreatic cancer stored at −80 °C were assayed for M65 and M30 levels. The median (inter-quartile range (IQR)) duration of plasma storage was 34 (23–57) months. Patients with metastatic disease (*n*=19) were found to have greater median (IQR) M65 levels (1145 (739–1698) U l^−1^) compared with the locally advanced (*n*=20; 748 (406–1150) U l^−1^) and resected (*n*=64; 612 (331–987) U l^−1^) patients (*P*=0.002). Elevated M65 levels were associated with poorer overall survival on univariate (*P*<0.001) but not multivariate (*P*=0.202) analysis. M65 concentrations also exhibited significant associations with concurrent serum–bilirubin levels (*P*<0.001) and the duration of plasma storage (*P*<0.001).

**Conclusions:**

Baseline plasma CK18 levels in pancreatic cancer are affected by the presence of obstructive jaundice and prolonged plasma storage. Clinical biomarker studies utilising serial CK18 levels are warranted in pancreatic cancer, provided consideration is given to these potentially confounding factors.

Pancreatic ductal adenocarcinoma is a leading cause of cancer deaths (http://www-dep.iarc.fr/) wherein, because of its characteristically late presentation, only 10–15% of patients present with resectable tumours (Alexakis *et al*, 2004). For the remaining 85–90% of patients presenting with inoperable disease, administration of systemic chemotherapy represents the most widely utilised palliative treatment modality (Yip *et al*, 2006). Conventional chemotherapy regimens incorporating either 5-FU or gemcitabine typically result in only modest improvement in overall survival (Sultana *et al*, 2007). One of the key challenges in accelerating improved cancer therapeutics is the identification of biomarkers to enable early objective assessment of tumour responses to chemotherapy (Kummar *et al*, 2007).

Necrosis of malignant and normal epithelial cells cause release of cytokeratin 18 (CK18) (Kramer *et al*, 2004), a type I intermediate filament protein and a component of the intracellular cytoskeleton (Fuchs and Weber, 1994). Caspase-mediated cleavage of the CK18 contributes to the degradation of the intracellular cytoskeleton if epithelial cells undergo apoptosis (Leers *et al*, 1999). Enzyme-linked immunosorbent assays (ELISAs) have been developed to measure circulating concentrations of both full-length CK18 (M65) and caspase-cleaved CK18 fragments (M30) (Kramer *et al*, 2004). Thus, the M65 ELISA reports necrotic and apoptotic epithelial cell death, whereas the M30 ELISA reports levels of epithelial apoptosis specifically (Kramer *et al*, 2004). Early studies suggest that these assays may have important clinical biomarker utility, as increased levels of circulating CK18 may be prognostic and/or predict tumour response to chemotherapy in a number of different solid tumours (Steele *et al*, 2008; Scott *et al*, 2009). Studies on specific tumour types include lung (Ulukaya *et al*, 2007; Hou *et al*, 2009), breast (Olofsson *et al*, 2007), prostate (Kramer *et al*, 2006), head and neck (Ozturk *et al*, 2009), colorectal (Ausch *et al*, 2009; Koelink *et al*, 2009) and testicular (de Haas *et al*, 2008) tumours. Although a recent study concluded that plasma levels of CK18 are a potential marker of tumour response in patients with advanced gastrointestinal malignancy (Scott *et al*, 2009) no previous studies have been reported in patients with pancreatic cancer.

The objective of this study was to characterise the baseline levels of circulating plasma CK18 concentrations using both the M65 and M30 ELISA assays in patients with pancreatic cancer. Correlations were also examined between CK18 and the circulating levels of carbohydrate antigen 19-9 (CA19-9), the only widely used biomarker in pancreatic cancer (Smith *et al*, 2008). Preoperative biliary stenting and concurrent liver function were also investigated in the interpretation of CK18 levels, as obstructive jaundice has been shown to be a confounding factor in proteomic studies (Yan *et al*, 2009) along with effect of plasma storage duration (Cummings *et al*, 2007; Greystoke *et al*, 2008).

## Materials and methods

### Patients and plasma collection

A total of 103 non-consecutive pancreatic adenocarcinoma patients with stored samples, treated at the Royal Liverpool University Hospital between 1997 and 2007 were identified from a prospective clinical database. All patients gave written consent for blood, tissue and clinical data to be used for research purposes.

All patients with obstructive jaundice routinely underwent biliary stenting at endoscopic retrograde cholangio-pancreatography. If endoscopic biliary decompression was unsuccessful, patients underwent percutaneous transhepatic cholangiography or combined procedures in order to decompress the biliary tree. In all, 64 patients then underwent a surgical resection for histologically confirmed pancreatic ductal adenocarcinoma (35 men and 29 women) with a median age of 68 (inter-quartile range (IQR)=61–73) years. A total of 39 patients had advanced pancreatic adenocarcinoma (22 men and 17 women) with a median age of 68 (IQR=58–77) years. The diagnosis of advanced disease was made at laparotomy in 30 of 39 (77%) patients. The remaining 9 cases (26%) were diagnosed as unresectable on initial imaging or at staging laparoscopy. In all, 15 of 64 patients undergoing resection went on to receive adjuvant therapy, chemotherapy in 13 and chemoradiotherapy in two. Patients with advanced cancer were managed at multiple referral institutions and data relating to palliative chemotherapy were available for 22 patients, 8 of whom had undergone chemotherapy. There was no significant change of the adjuvant or palliative therapy protocols during the study period.

Plasma was collected on the day before surgical intervention and stored at −80°C. Plasma was collected at the time of diagnosis in the nine patients managed nonoperatively. The median time interval for plasma storage (from the date of plasma collection to the date of assay) was 34.1 (IQR=22.7–57.0) months. Preoperative serum CA19-9 and concurrent bilirubin levels were also retrieved, along with details regarding resected histopathological tumour characteristics and patient survival data. Overall survival times were calculated from the date of plasma sampling to the date of death.

### Detection of M65 and M30 antigens

The M65 and M30 (Apoptosense) ELISA kits (Peviva, Ströskarlsvägen, Sweden) were used for the plasma biomarker analyses according to previously described methods and according to good clinical laboratory practice (GCLP, Cummings *et al*, 2005). An apoptotic index was calculated from the ratio of caspase-cleaved CK18 as a proportion of overall CK18 (M30 divided by M65) as previously described (Kramer *et al*, 2004).

### Preparation of tissue microarray (TMA)

The original haematoxylin and eosin (H&E) stained histopathology slides from resected cases were retrieved and reviewed by a consultant pathologist (FC). Slides were marked in order to target cancer cores for subsequent sampling from stored paraffin-embedded tissue blocks. It was ensured that targeted areas were away from resection margins to avoid diathermied tissue. Duplicate cores taken from three areas within each tissue block were sampled for each patient. Normal tissue from kidney, colon and duodenum were used for orientation cores. Five control blocks from chronic pancreatitis specimens were also used in the TMA.

The marked slide and paraffin-embedded tissue block were aligned so that the targeted area of tumour on the donor block could be determined. Using a manual tissue arrayer (Beecher Instruments, Sun Prairie, WI, USA), 0.6 mm diameter tissue cores were taken to a depth of 4 mm from the targeted area of tumour in the donor block. The tissue cores were placed into the recipient paraffin block 1 mm apart. The TMA blocks were cut using a Leica Reichert-Jung BIOCUT 2035 microtome (Leica Biosystems, Wetzlar, Germany) in 5 μm sections. Two 5 μm sections were cut from each block and mounted on X-tra adhesive Surgipath slides in preparation for immunohistochemistry (IHC).

### Detection of cleaved caspase 3

Cleaved caspase 3 was assessed by immunohistochemistry using a validated method to GCLP. Tissue microarray sections were deparaffinised, re-dehydrated and antigen retrieval was carried out using microwaves for 25 min in citric acid buffer (10 mM, pH 6.0). Sections were then blocked by immersing in a 0.3% hydrogen peroxide solution for 30 min to remove exogenous and endogenous peroxidase. The slides were blocked using 10% casein solution for 1 h to remove background staining. Slides were then incubated overnight with the primary antibody (Cell Signaling Technology, Boston, MA, USA; ref: #9661S) at 4 °C in a humidified tray. Slides were then washed in PBS and goat anti-rabbit (Vectastain ABC kit from Vector Laboratories, Burlingame, CA, USA; ref: PK-4001) secondary antibody was added for 30 min. The slides were washed with PBS and ABC kit (Envision kit, Vector Laboratories) was applied (according to manufacturer's instructions). Sections were washed in PBS before visualization of signal using DAB reagent. Sections were then counterstained with haematoxylin before mounting.

### Immunohistochemical scoring

**Figure 1:**
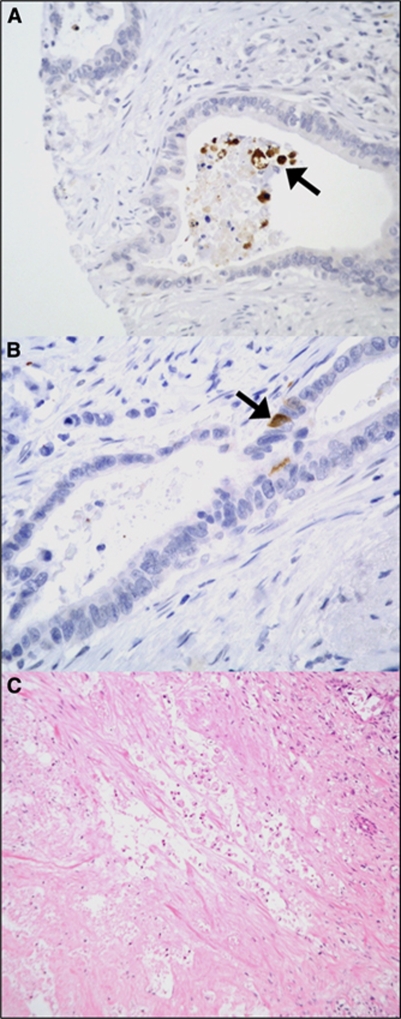
(**A**) Example of shed cells within a cancer gland exhibiting positive staining for activated caspase-3 on immunohistochemistry (arrowed) in a core of pancreatic ductal adenocarcinoma. (**B**) Example of sporadic nature of activated caspase-3 staining within the malignant ductal epithelium. (**C**) Haematoxylin and eosin (H&E) stained slide of pancreatic ductal adenocarcinoma exhibiting widespread tumour necrosis.

The activated caspase-3 immunohistochemistry was scored by a consultant pathologist (FC) according to the presence of positive staining in the intraluminal shed cells within cancer glands (Figure 1A). Only tissue cores exhibiting one or more shed cells were scored. Cases were classified as either positive or negative based on the proportion of positively stained cells present. Cases where more than 50% of shed cells exhibited positive staining in a majority of cancer cores were classified as positive. Owing to the infrequent staining exhibited in the intact malignant epithelium (Figure 1B), no attempt to score the epithelial staining was made.

The original H&E stained histopathology slides for the resected pancreatic specimens were also evaluated by the same consultant pathologist (FC) to make a quantitative assessment of tumour necrosis on microscopic examination. Cases were allocated a percentage score (0, 1, 5, 10, 25 or 50%) according to the proportional area of tumour material exhibiting microscopic features of necrosis (Figure 1C). Areas of fibrosis, sclerosis and autolysis on microscopy were not included as part of this assessment. The pathologist was blinded to the ELISA results and survival data during scoring of both the activated caspase-3 immunohistochemistry and the microscopic assessment of tumour necrosis.

### Statistical analysis

Continuous data were described using median values, IQR and 95% confidence intervals (CIs) with Mann–Whitney *U*-testing or Kruskal–Wallis testing for non-parametric comparisons of continuous data. Categorical data were analyzed using the *χ*^2^ test. Spearman's rank correlation was used to analyse the relationship between two individual continuous variables. CA19-9 was normalised for regression analyses by logarithmic transformation (log_e_CA19-9). Survival data were analysed using the Kaplan–Meier method with log-rank testing for the comparison of survival curves. Cox's proportional hazards regression was used for multivariate analysis. Prognostic variables of univariate significance were selected for inclusion in the multivariate model. A *P*-value of 0.05 was considered significant.

## Results

**Figure 2:**
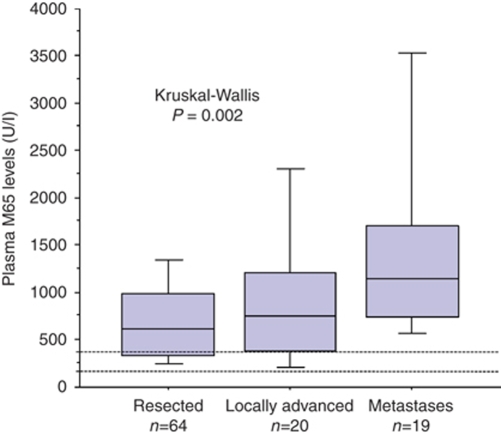
Box plot illustrating the effect of tumour resectability and presence of metastasis on plasma M65 levels. The dotted lines represent the inter quartile range (IQR) reference values of 191 and 339 U l^−1^ for healthy controls (Greystoke *et al*, 2008).

Of the 103 patients analysed, plasma M65 levels were measureable for 102 and M30 levels measureable for 98 patients. Both assays were measureable for 97 patients. Table 1 gives details of the demographic and clinicopathological data for the overall patient group. The median (IQR) plasma M65 level recorded in the overall patient group was 724 (411, 1188) U l^−1^, whereas the median plasma M30 level was 212 (149, 327) U l^−1^ and the M30 : M65 ratio was 0.32 (0.24, 0.49). The median (IQR) reference values for M30 and M65 were determined in 82 healthy controls (30 men and 52women) with a median age of 45 (35, 55) years and were 198 (155, 317) U l^−1^ and 240 (191, 339) U l^−1^, respectively (Greystoke *et al*, 2008). The three clinical groups of resected, locally advanced not resected and metastasis groups may be considered as demonstrating increasing tumour burden and so the results for the M30 and M65 levels and the M30 : M65 ratios are shown by these groups in Table 2. The median (IQR) M65 levels significantly increased with increasing degrees of tumour burden being 1145 (572, 1479) U l^−1^ for patients with metastatic disease compared with 748 (406, 1150) U l^−1^ in patients with locally advanced non-resectable disease and 612 (331, 987) U l^−1^ in patients who had resection (Kruskal–Wallis, *P*=0.002; Table 2 and Figure 2).

### Confounding factors

Carbohydrate antigen 19-9 (*P*=0.007) and bilirubin (*P*=0.004) levels were also associated with the increasing tumour burden (Table 2). Plasma M65 levels were found to exhibit a strong correlation with concurrent serum–bilirubin levels (Spearman, *ρ*=0.439, *P*<0.001) but not CA19-9 levels. A significant correlation between M65 levels and the duration of plasma storage overall was found (Spearman, *ρ*=0.487, *P*<0.001) and the period of plasma storage was also unexpectedly found to be significantly longer in the group with metastases (*P*=0.007).

### Plasma CK18 levels and survival

**Figure 3:**
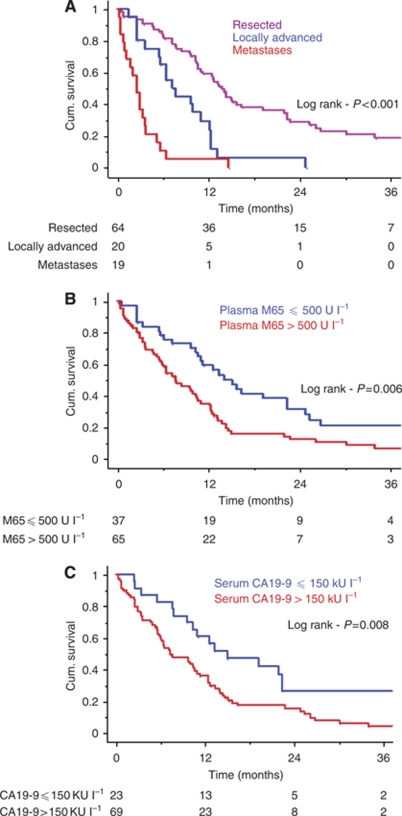
Kaplan–Meier curves to illustrate survival trends in overall patient group. (**A**) The expected pattern of worsening survival associated with more advanced disease was observed. The survival curves according to plasma M65 levels (**B**) were comparable with those seen for serum carbohydrate antigen (CA)19-9 levels (**C**) in the overall patient group.

Resectability, the presence of metastases and M65, Ca19-9 and bilirubin levels were all significantly associated with survival (all *P*<0.001) on univariate analyses (Table 3 and Figure 3). Resected patients had a median survival of 13.9 (95% CI=11.0–16.4) months compared with 7.3 (95% CI=5.5–11.0) months for locally advanced tumours and 2.5 (95% CI=1.2–3.6) months for patients with metastatic disease (log rank, *P*<0.001). Increasingly elevated plasma M65 levels were associated with poorer survival in the overall patient group on univariate analysis both as a continuous (Cox, *P*<0.001; Table 3) and dichotomised variable (log rank, *P*=0.007; Figure 3B). Neither M30 levels (Cox, *P*=0.052) nor the M30 : M65 ratio (Cox, *P*=0.096) were significant univariate prognostic variables in the overall patient group. The results of a multivariate analysis including M65 levels, tumour resectability, CA19-9 and bilirubin are shown in Table 3. In this model the only independent significant prognostic factors were resectability and the presence of metastases (both *P*<0.001).

### Histopathological assessment of necrosis and apoptosis in resected cases

There was no significant relationship between plasma CK18 levels and tumour histology. The original histopathology slides with corresponding paraffin-embedded tumour material were available in 58 of 64 resected patients (91%). A total of 17 patients (29%) exhibited significant areas of tumour necrosis (i.e. ⩾5%) on microscopic assessment. There was no significant correlation between plasma M65 levels and the histological percentage score for microscopic tumour necrosis (Spearman, *ρ*=0.084, *P*=0.531). After the preparation of TMA, a total of 53 out of 58 cases had one or more evaluable cores containing cancer. In all, 45 cases exhibited shed cells, which could be evaluated for activated caspase-3 staining. Of these 45 cases, 25 (56%) had positive staining and 20 (44%) were negative. There was no significant difference in the median plasma M30 : M65 ratio between positive (0.37 (IQR=0.26–0.53)) and negative cases (0.36 (IQR=0.26–0.48)) for activated caspase-3 immunostaining (Mann–Whitney, *P*=0.478).

## Discussion

Cytokeratin 18 is a type I intermediate filament protein component of the intracellular cytoskeleton in epithelial cells (Fuchs and Weber, 1994). Increasing evidence exists to suggest that cytokeratins have an important role during epithelial apoptosis. Degradation of type I cytokeratins (including CK18) by activated caspases 3, 7 and 9 with consequent loss of mechanical integrity of the cell occurs during the execution phase of apoptosis to facilitate cell shrinkage and formation of apoptotic bodies (Leers *et al*, 1999; Schutte *et al*, 2004). During this process, CK18 is cleaved at two specific sites along the non-helical linker region of the protein (Asp238 and Asp396) exposing the Asp396 neo-epitope with the subsequent release of cleaved cytokeratin fragments into the extracellular space. Detection of this Asp396 neo-epitope by the M30 ELISA is, therefore, only possible following caspase-dependent apoptosis. It has been postulated that necrosis of epithelial cells releases full-length cytokeratins into the extracellular space following breakdown of the cell membrane (Bodenmüller *et al*, 1994). *In vitro* studies have shown that induction of necrosis in a human breast epithelial cell line is associated with a significant increase in the concentration of extracellular CK18, as recorded by the M65 ELISA (Kramer *et al*, 2004).

This study represents the first attempt to characterise circulating levels of CK18 in patients with pancreatic cancer in order to assess its clinical biomarker potential. The results from the M30 and M65 ELISA assays showed significant variability between patients with regard to the baseline levels of both full-length CK18 (M65) and the proportion of caspase-cleaved CK18 (M30 : M65). Circulating total CK18 concentrations in this study were relatively high compared with prostate (Kramer *et al*, 2006) and breast (Olofsson *et al*, 2007) cancer and comparable with those of other gastrointestinal malignancies (Scott *et al*, 2009) and non-small-cell lung cancer (Hou *et al*, 2009). In keeping with the other malignant tumour types (Ulukaya *et al*, 2007; Hou *et al*, 2009; Koelink *et al*, 2009), elevated CK18 levels were associated with poorer survival in the overall patient group on univariate analysis, but in this series failed to reach significance on multivariate analysis.

There was no significant association between plasma M65 levels and the histopathological assessment of tumour necrosis. This finding may implicate additional factors other than intrinsic tumour biology having an important confounding effect on circulating M65 concentrations. A marked correlation was seen between the concurrent bilirubin levels and circulating CK18 levels. This observation is likely to be explained on the basis that obstruction of the main bile duct with consequent dilatation and epithelial disruption directly influences the balance of proliferation and cell death within the biliary epithelium (Lesage *et al*, 2001; Alpini *et al*, 2003). Low-grade cholangitis is quite common following biliary stenting and may also represent an additional confounding factor, as both generalised sepsis (Roth *et al*, 2004) and cholangitis (Yagmur *et al*, 2007) raise circulating CK18 concentrations. Other studies have demonstrated significant disturbances in circulating CK18 in patients with chronic liver disease (Hetz *et al*, 2007; Yagmur *et al*, 2007).

Information on the long-term antigen stability of the M30 and M65 ELISAs in stored human plasma is limited. A previous study (Cummings *et al*, 2007) in 20 patients with cancer showed no significant degradation of the M65 antigen after 2 years of storage at −80^o^C although the M30 antigen exhibited increased values with extended storage in a proportion of patients and confirmed in a more recent study (Greystoke *et al*, 2008). The results from this, much larger, study have shown that antigen levels exhibit a trend towards more elevated values when stored over a longer period.

Appropriate pre-clinical validation of potential cancer biomarkers is essential before their utilisation in either routine clinical practice or trial settings (Cummings *et al*, 2008). This study highlights the fact that the pathophysiology of pancreatic cancer presents a number of different challenges with regard to the analysis of blood–borne biomarkers. Studies utilising serial CK18 measurements to determine tumour responses to cytotoxic therapy in pancreatic cancer should give adequate consideration to the potential confounding factors of concurrent obstructive jaundice and the duration of sample storage.

## Figures and Tables

**Table 1:** Demographics and clinicopathological data

**Total number of patients**	**103**
Median age (IQR) (years)	68 (60–74)
Male : Female	57 : 46
Resected cases:	64
Median tumour size (IQR) (mm)	30 (22–38)
T1/T2 : T3/T4	11 : 52
N0 : N1	10 : 52
Well/moderate/poor differentiation	10/35/18
R0 : R1	19 : 45
Unresected cases:	39
Locally advanced	20
Metastatic	19
Median M65 (IQR) (U l^−1^)	724 (411–1188)
Median M30 (IQR) (U l^−1^)	212 (149–327)
Median M30 : M65 ratio (IQR)	0.32 (0.24–0.49)
Median preoperative CA19-9 (IQR) (kU l^−1^)	463 (151–1265)
Median preoperative bilirubin (IQR) (*μ*mol l^−1^)	23 (12–57)
Median duration of plasma storage (IQR) (months)	34 (23–57)

CA=carbohydrate antigen; IQR=interquartile range.

The median (IQR) time interval from the date of preoperative CA19-9 estimation to the date of plasma sampling was 28 (19, 38) days. The median (IQR) time interval for liver function tests was 1 (1, 2) day. Histological data was incomplete for one patient. Preoperative CA19-9 and bilirubin data were incomplete for 11 and 14 patients, respectively. The median (IQR) values for M30 and M65 in 82 healthy controls were 198 (155, 317) U l^−1^ and 240 (191, 339) U l^−1^, respectively (Greystoke *et al*, 2008).

**Table 2:** Parameters according to resectability and presence of metastases

	**Resected cases (*N*=64)**	**Locally advanced unresected cases (*N*=20)**	**Metastatic unresected cases (*N*=19)**	***P*-value**
Median age (IQR) (years)	68 (61–73)	70 (64–77)	65 (57–75)	0.406
Male : Female	35 : 29	8 : 12	14 : 5	0.105^*^
Median M65 (IQR) (U l^−1^)	611 (331–987)	748 (406–1150)	1145 (739–1698)	0.002
Median M30 (IQR) (U l^−1^)	192 (147–311)	215 (142–348)	324 (224–453)	0.058
Median M30 : M65 ratio (IQR)	0.39 (0.24–0.50)	0.29 (0.21–0.40)	0.28 (0.25–0.33)	0.184
Median preoperative CA19-9 (IQR) (kU l^−1^)	356 (81–893)	416 (217–2310)	1628 (581–3312)	0.007
Median preoperative bilirubin (IQR) (*μ*mol l^−1^)	22 (12–37)	23 (11–51)	99 (21–204)	0.004
Median duration of plasma storage (IQR) (months)	29 (21–42)	43 (29–60)	106 (32–114)	0.003

CA=carbohydrate antigen; IQR=inter quartile range.

Quoted *P*-values for Kruskal–Wallis test. ^*^χ^2^ *P*-value.

**Table 3:** Survival analysis

	**Univariate analysis**			**Multivariate analysis (n=82)**		
	**Hazard ratio (95% CI)**	***χ* ^2^**	***P***	**Hazard ratio (95% CI)**	***χ* ^2^**	***P***
Tumour resectability:	—	50.85	<0.001		24.73	<0.001
Resected (*n*=64)	—	—	—	—	—	—
Locally advanced (*n*=20)	2.742 (1.567–4.796)	12.50	<0.001	2.579 (1.340–4.962)	8.05	0.005
Metastases (*n*=19)	8.489 (4.678–15.405)	49.50	<0.001	6.533 (3.048–14.000)	23.29	<0.001
Plasma M65	1.001 (1.000–1.001)	15.55	<0.001	1.000 (1.000–1.001)	1.63	0.202
LogCA19-9	1.235 (1.095–1.394)	11.72	<0.001	1.130 (0.964–1.325)	2.26	0.133
Bilirubin	1.003 (1.001–1.005)	11.01	<0.001	0.999 (0.995–1.003)	0.44	0.507
Chemotherapy	0.864 (0.505–1.476)	0.29	0.592	—	—	—

CA=carbohydrate antigen; CI=confidence interval.

^*^Plasma M65 levels, CA19-9 and bilirubin were modelled as continuous prognostic covariates. Quoted hazard ratios for continuous variables signify the relative hazard associated with each incremental increase in the covariate value by 1 U. Logarithmic transformation of CA19-9 results was undertaken to normalise for Cox's regression because of the wide range of preoperative CA19-9 results recorded in the overall patient group (from 1–90 000 kU l^−1^).
